# Christians and Buddhists Are Comparably Happy on Twitter: A Large-Scale Linguistic Analysis of Religious Differences in Social, Cognitive, and Emotional Tendencies

**DOI:** 10.3389/fpsyg.2019.00113

**Published:** 2019-02-06

**Authors:** Chih-Yu Chen, Tsung-Ren Huang

**Affiliations:** ^1^Department of Psychology, National Taiwan University, Taipei, Taiwan; ^2^Center for Research in Econometric Theory and Applications, National Taiwan University, Taipei, Taiwan

**Keywords:** religion, Christianity, Buddhism, sociolinguistics, social interaction, cognition, emotions

## Abstract

Are different religions associated with different social, cognitive, and emotional tendencies? Although major world religions are known to encourage social interactions and help regulate emotions, it is less clear to what extent adherents of various religions differ in these dimensions in daily life. We thus carried out a large-scale sociolinguistic analysis of social media messages of Christians and Buddhists living in the United States. After controlling for age and gender effects on linguistic patterns, we found that Christians used more social words and fewer cognitive words than Buddhists. Moreover, adherents of both religions, similarly used more positive than negative emotion words on Twitter, although overall, Christians were slightly more positive in verbal emotional expression than Buddhists. These sociolinguistic patterns of actual rather than ideal behaviors were also paralleled by language used in the popular sacred texts of Christianity and Buddhism, with the exception that Christian texts contained more negative and fewer positive emotion words than Buddhist texts. Taken together, our results suggest that the direct or indirect influence of religious texts on the receivers of their messages may partially, but not fully, account for the verbal behavior of religious adherents.

## Introduction

Religion is an important system of beliefs and values that guides people’s mental processes and behaviors (Cohen and Hill, 2007). In 2010, 84% of the 6.9 billion people around the world considered themselves religious, and the percentage will grow to 86.8% of a projected 9.3 billion people (Hackett et al., 2015). Being religious has been shown by sociologists and psychologists to be beneficial in many respects (Chatters, 2000; Emmons and Paloutzian, 2003; Ekman et al., 2005). For example, religion can provide explanations about uncertain circumstances (e.g., natural disasters, death, and poverty) and guidance for living a meaningful life (Sibley and Bulbulia, 2012; Oishi and Diener, 2014; Norenzayan, 2016). In addition, religiosity was found to buffer against anxiety, minimize experiences of errors, and reduce recurrence of depression (Inzlicht et al., 2009; Inzlicht and Tullett, 2010; Miller et al., 2012).

Even though there are approximately 10,000 religious traditions around the world, psychological studies of religion have more often examined differences between religious vs. nonreligious groups rather than differences among various religious groups. Fewer studies have quantitatively compared psychological effects among religions. Their conclusions are often drawn from self-reported survey data (e.g., Saroglou et al., 2004; Tsai et al., 2007; Cohen, 2015), which, particularly in the case of emotional assessment, could be subject to response biases (Wojcik et al., 2015).

To avoid making inferences from biased self-report responses, two earlier studies carried out large-scale observations of *spontaneous* verbal behavior of religious adherents on social media platforms to investigate religious differences. Examining Twitter feeds, Ritter et al. (2014) found that Christians linguistically appeared happier, more socially connected, and less analytical than atheists. On Facebook, Yaden et al. (2018), similarly observed that religious individuals (86% Christians) used more social and positive emotion words, while nonreligious individuals used more cognitive and negative emotion words.

These linguistic patterns in earlier studies were discovered using a computational linguistics program—Linguistic Inquiry and Word Count (LIWC; Pennebaker et al., 2015). LIWC calculates the percentages of words in a text belonging to 125 predefined word categories. These word categories can be standard linguistic categories, such as Articles (a, an, the), or categories related to psychological states and processes, such as Affect and Social Processes. Importantly, these LIWC categories have internal reliability and external validity (Pennebaker et al., 2015) and can be related to real-world outcome measures (for a review, see Tausczik and Pennebaker, 2010).

The social, cognitive, and emotional tendencies observed by Ritter et al. (2014) and Yaden et al. (2018) in the language of Christians on social media appear to manifest Christians’ psychological tendencies in the real world. Christians’ more frequent use of social and positive emotion words is consistent with previous findings that religious individuals have fewer negative emotions and better well-being. This finding is in part due to engaging in more social interactions (Lim and Putnam, 2010; Diener et al., 2011; Gebauer et al., 2012). However, it remains unclear whether these psychologically beneficial effects are Christian-specific or general religious effects. Therefore, the present study aimed to compare these social, cognitive, and emotional tendencies across different religions.

Specifically, we used LIWC to compare Western-originated Christianity with Eastern-originated Buddhism. These two major world religions differ distinctly in many respects, such as their central doctrines (Nicholson, 2016), ideal affect (Tsai et al., 2007), and attitudes towards outgroups (Clobert et al., 2015). Because these religious differences in beliefs, values, and attitudes may not directly translate to behavioral differences (Ajzen, 2005), we did not have a strong hypothesis regarding the differences in verbal behavior between Christians and Buddhists.

Despite being exploratory in nature, the present study does have clear predictions about the relationship between social, cognitive, and emotional tendencies regardless of religions. Because social interaction usually increases happiness for individuals in either independent or interdependent cultures (Lu et al., 2001; Kitayama et al., 2010; Hitokoto and Uchida, 2015) and excessive thinking often leads to negative emotions regardless of cultural backgrounds (e.g., Dore et al., 2015; Kaiser et al., 2015), we expected to see a positive relationship between the use of *social* and *positive emotion* words and a negative relationship between the use of *cognitive* and *negative emotion* words.

To examine the extent to which religious adherents act in accord with their religions, we also applied the same linguistic analysis to Christian and Buddhist sacred texts under the assumption that these religious texts reflect the foundational ideas, worldviews, and emotional tones of each religion (Whissell and Dewson, 1986; Tsai et al., 2007). Because these religious texts may influence the mind and behavior of the receivers of their messages, either directly through reading or indirectly through exposure to preaching by others (Bushman et al., 2007), we expected that any sociolinguistic patterns found among Christians and Buddhists would partially mirror those found in Christian and Buddhist sacred texts.

## Materials and Methods

### Participants

In the literature, a sample size larger than 120 participants per religious group was sufficient to show significant group differences in emotions (e.g., Tsai et al., 2007). In the present study, 10,000 Christians and 10,000 Buddhists in the United States were randomly sampled as participants. Participants were followers of the two most popular Christian and Buddhist accounts on Twitter, and their religious affiliations were designated according to which account they followed.

To control for gender and age effects (see Supplementary Figures S1–S6; Charles and Carstensen, 2010; Stone et al., 2010) in our comparison between the two religions, we inferred participants’ genders and ages from their social media messages—Tweets. The lexicon-based predictive model we used could reach a prediction accuracy of 0.82 for age and 0.90 for gender using only 100 Tweets from each person (see Sap et al., 2014 and Supplementary Materials for details). According to our estimates of participants’ gender and age, we then divided our participant pool into eight strata (the strata were a combination of females vs. males and four age groups: 18–24, 25–34, 35–44, and 45–54 years of age), and sampled 1,250 people from each stratum of each religion.

### LIWC Categories

We collected 100 English Tweets (∼1,140 words) posted by each participant and used LIWC to compute the proportion of words related to social, cognitive, and affective processes in each user’s Tweets (see Supplementary Table S1). Note that some words may be shared across different LIWC categories, many of which have a hierarchical relationship. For example, “we”, “you”, and “they” are words listed in both the Personal Pronouns and Social Processes categories, while the Sad category is a subcategory of Negative Emotion, which is in turn a subcategory of Affect. Therefore, different LIWC output measures are not statistically independent of each other.

To provide converging evidence for our conclusions, we also examined several other LIWC categories that are either subcategories of or closely related to the categories of social, cognitive, and affective processes. For instance, personal pronouns were analyzed because they reveal how an individual references others in social interactions and outside of them (Tausczik and Pennebaker, 2010). An analytic index was computed to supplement the analysis on cognitive processes because it reveals the degrees to which an individual relies on formal/logical than informal/narrative thinking (Pennebaker et al., 2014; Jordan and Pennebaker, 2017).

### Sacred Texts

It is worth noting that the measured percentage of a LIWC category in a text is a function of text length. For example, the sentence “I feel happy” could be seen as having 33% positive words in a 3-word chunk or, instead, having one 100% positive words and two 0% positive words if the chunk unit was only one word. Therefore, text length should be controlled for in the comparisons between Tweets and sacred texts.

In the analysis of English sacred texts, the most popular Protestant Bible books and Buddhist sacred texts were selected (see Supplementary Tables S2, S3) and divided into excerpts of 1,000 words to be on par with the number of analyzed words for each Twitter participant. In total, our LIWC-analyzed Bible and Buddhist texts consisted of 305 and 144 excerpts, respectively.

## Results

In our statistical comparisons, the nonparametric Kolmogorov–Smirnov (KS) test was used to assess whether two distributions significantly differed from each other, and the Cohen’s d was used to determine the effect size of the difference between two distributions. To infer the direction of such a difference, all our KS tests are one-tailed tests and all of our reported significance levels of *p* < 0.001 hold true even with Bonferroni corrections for multiple comparisons on the 125 LIWC categories. As for effect sizes, d = 0.2, 0.5, 0.8 are considered as a small, medium, and large effect sizes, respectively (Cohen, 1992).

### Social Processes

**FIGURE 1:**
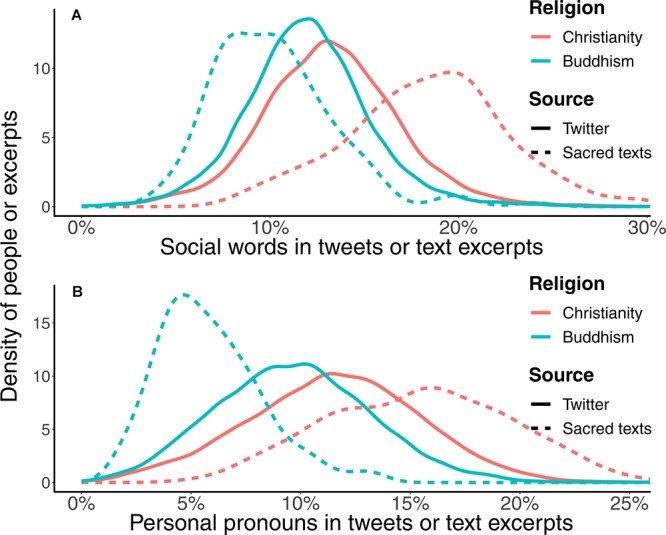
Distributions of the relative frequencies of **(A)** social words and **(B)** personal pronouns in user’s Tweets of different religions and in different religious texts. Christian Twitter users used more social words and personal pronouns than Buddhists. There were also more social words and personal pronouns in the Bible than in the Buddhist sacred texts.

Linguistically, Christians appeared more social than Buddhists, as shown in Figure 1. Specifically, Christians used more social words than Buddhists on Twitter (*KS D* = 0.17, *p* < 0.001, *Cohen’s d* 95% *CI*: [0.32, 0.37]), such as words regarding family (*d* = 0.40), friends (*d* = 0.37), female references (*d* = 0.21), and male references (*d* = 0.55). Closely related to social processes, personal pronouns were also used more frequently by Christians than by Buddhists on Twitter (e.g., me, she, and we’ll; *KS D* = 0.22, *p* < 0.001, *Cohen’s d* 95% *CI*: [0.45, 0.51]).

The observed lexical patterns were also found in the comparison between Christian and Buddhist sacred texts; social words and personal pronouns were used more often in the Bible than in the Buddhist texts (social words: *KS D* = 0.75, *p* < 0.001, 95% *Cohen’s d* 95% *CI*: [1.83, 2.31]; personal pronouns: *KS D* = 0.85, *p* < 0.001, *Cohen’s d* 95% *CI*: [3.76, 4.43]).

### Cognitive Processes

**FIGURE 2:**
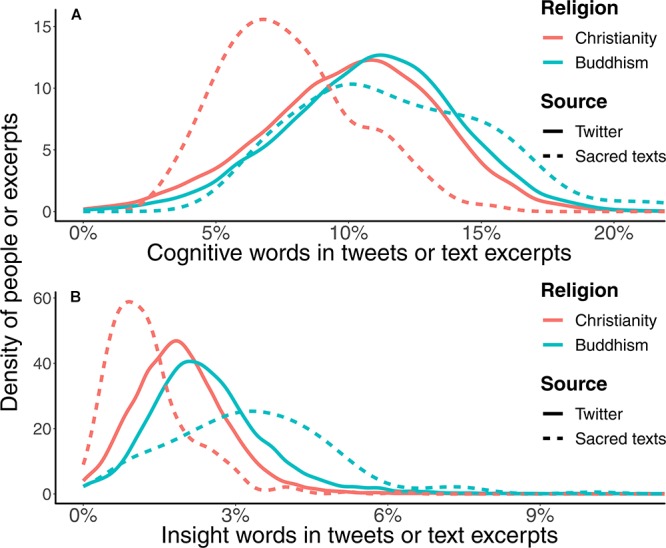
Distributions of the relative frequencies of **(A)** cognitive words and **(B)** insight words in user’s Tweets of different religions and in different religious texts. Buddhist Twitter users used more cognitive words and insight words than Christians. In the sacred texts, the Buddhist texts also contained more cognitive words and insight words than the Bible.

Linguistically, Buddhists appeared to reason more than Christians, as shown in Figure 2. Specifically, Buddhists used more cognitive words than Christians on Twitter (*KS D* = 0.09, *p* < 0.001, *Cohen’s d* 95% *CI*: [0.19, 0.24]), such as words indicating insight (*d =* 0.47), causation (*d =* 0.40), discrepancy (*d =* 0.11), tentativeness (*d =* 0.05), certainty (*d =* 0.11), and differentiation (*d =* 0.04). Closely related to cognitive processes, the overall analytic index score was higher for Buddhist than Christian Tweets (*KS D =* 0.16, *p <* 0.001, *Cohen’s d* 95% *CI*: [0.37, 0.42]).

The observed lexical patterns were also found in the comparison between Christian and Buddhist sacred texts; the Buddhist texts contained more cognitive and insight words than the Bible (cognitive: *KS D* = 0.48, *p* < 0.001, *Cohen’s d* 95% *CI*: [1.16, 1.59]; insight: *KS D* = 0.63, *p* < 0.001, *Cohen’s d* 95% *CI*: [1.05, 1.48]). Moreover, Buddhist texts had higher analytic index scores than the Bible (*KS D* = 0.62, *p* < 0.001, *Cohen’s d* 95% *CI*: [2.19, 2.70]).

### Affective Processes

#### Relationship Between Social, Cognitive, and Emotional Tendencies

Consistent with our predictions, regardless of religion, the 20,000 participants in our study showed a lexical tendency for the relative frequencies of social words to be positively correlated with the relative frequencies of positive emotion words (Pearson’s r = 0.22, *p* < 0.001). Additionally, the relative frequencies of cognitive words were positively correlated with those of negative emotion words (Pearson’s *r* = 0.36, *p* < 0.001) and negatively correlated with those of positive emotion words (Pearson’s *r* = -0.15, *p* < 0.001).

#### Comparison of Emotional Tendencies Across Religions

**FIGURE 3:**
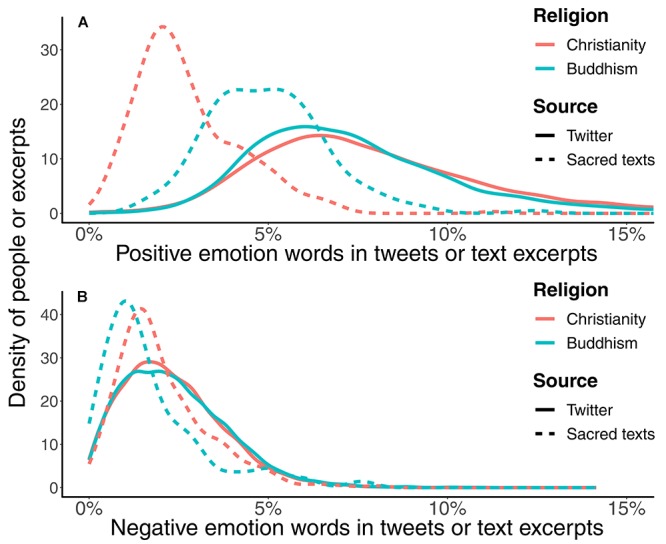
Distributions of the relative frequencies of **(A)** positive and **(B)** negative emotion words in user’s Tweets of different religions and in different religious texts. Christians were slightly more positive than Buddhists in verbal emotional expression. In contrast, the Bible contained more negative emotion words while the Buddhist sacred texts contained more positive emotion words.

In terms of verbal emotional expressions, the results in Figure 3 show that Christians used slightly more positive emotion words than Buddhists (e.g., glad, happy, and cheer; *KS D* = 0.08, *p* < 0.001, *Cohen’s d* 95% *CI*: [0.16, 0.22]), and Buddhists used slightly more negative emotion words than Christians (*KS D* = 0.02, *p* = 0.0072, 95% *Cohen’s d* 95% *CI*: [0.01, 0.06]), such as words expressing anxiety (*d* = 0.27) and anger (*d* = 0.05).

The patterns of emotional tones in Christian and Buddhist sacred texts appeared to be opposite to those observed in Christian and Buddhist Tweets. Specifically, there were more positive and fewer negative emotion words in the Buddhist texts than in the Bible (positive: *KS D* = 0.59, *p* < 0.001, *Cohen’s d* 95% *CI*: [1.21, 1.65]; negative: *KS D =* 0.23, *p* = 0.000049, *Cohen’s d* 95% *CI*: [-0.43, -0.03]).

#### Comparison of Emotional Tendencies Within Each Religion

**FIGURE 4:**
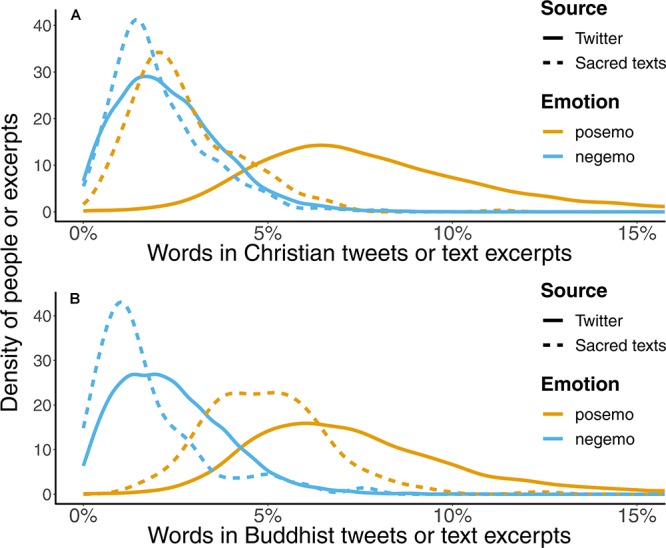
Distributions of the relative frequencies of emotional words in **(A)** Christian texts and **(B)** Buddhist texts. Both Christian and Buddhist Twitter users used more positive than negative emotion words. Similarly, both Christian and Buddhist sacred texts also contained more positive than negative emotion words.

When the number of positive and negative emotion words was compared within each religion rather than across religions, the overall emotional tones of both sacred texts and Tweets were then, similarly positive, as shown in Figure 4. More precisely, the sacred texts of both religions overall contained more positive than negative emotion words (Christian: *KS D* = 0.28, *p* < 0.001, *Cohen’s d* 95% *CI*: [0.35, 0.67]; Buddhist: *KS D* = 0.78, *p* < 0.001, 95% *Cohen’s d* 95% *CI*: [1.75, 2.32]) as did Tweets from adherents of both religions (Christian: *KS D* = 0.81, *p* < 0.001, *Cohen’s d* 95% *CI*: [1.93, 2.00]; Buddhist: *KS D* = 0.79, *p* < 0.001, *Cohen’s d* 95% *CI*: [2.02, 2.09]).

## Discussion

In summary, our exploratory analysis found that adherents of the two religions exhibited detectable linguistic differences regarding their cognitive, social, and emotional tendencies; Christians used slightly more social and positive emotion words whereas Buddhists used slightly more cognitive and negative emotion words. Moreover, these sociolinguistic patterns found in Christians and Buddhists to some extent mirrored those found in Christian and Buddhist sacred texts, with the exception of emotional tones. Our results replicate the previous findings that linguistically Christians appear happier, more socially connected, and less analytical than nonreligious individuals on social media (Ritter et al., 2014; Yaden et al., 2018) and further clarify that these psychological tendencies are Christian-specific rather than general religious effects.

Linguistically, Christians appeared to be more social than Buddhists. This result could be due to higher levels of extraversion in Christians than Buddhists (Tsai et al., 2007) and higher frequency of social events in Christian churches than in Buddhist temples or other gathering places. Being social is also encouraged in the Bible, as evidenced by the verses from Romans 12:15–18 (New International Version): ”*Rejoice with those who rejoice; mourn with those who mourn. Live in harmony with one another. Do not be proud, but be willing to associate with people of low position. Do not be conceited. Do not repay anyone evil for evil. Be careful to do what is right in the eyes of everyone. If it is possible, as far as it depends on you, live at peace with everyone.*” Christians may thus value and like to talk about their social events.

Linguistically Buddhists appeared to reason more than Christians. Buddhists may cultivate a habit of actively and causally reflecting on every aspect of their lives because causality is the central philosophy of Buddhism, which emphasizes the interdependence among events and understanding of how a chain of causes leads to suffering in everyday life (Kalupahana, 1986). Moreover, to increase awareness and thinking of the causes of suffering (e.g., egocentricity and belief in permanence), some Buddhist texts deliver teachings in an unconventional and sometimes convoluted manner. Here is one such example excerpted from the Heart Sutra (Thich, 2014): “*Listen Sariputra, this Body itself is Emptiness and Emptiness itself is this Body. This Body is not other than Emptiness and Emptiness is not other than this Body. The same is true of Feelings, Perceptions, Mental Formations, and Consciousness.*” Buddhists may become accustomed to and even adopt such a thinking style in their spoken and written languages.

As for emotional expressions, both Christians and Buddhists used more positive than negative emotion words, as did all of the religious texts we analyzed. Such a positivity bias is a common pattern in the analyses of English texts using LIWC. On Twitter, the mean percentages of positive and negative emotion words were previously reported to be 5.48% and 2.14%, respectively (Pennebaker et al., 2015). Our observed mean percentages of positive and negative emotion words were 8.20% and 2.34% in Christian Tweets, and 7.51% and 2.39% in Buddhist Tweets, respectively. The emotional positivity may be amplified by both religions. For example, both Christians and Buddhists were taught to value and experience positive emotions more than negative emotions (Tsai et al., 2007), and references to happiness, but not other emotions, were found to increase in the Hebrew Bible over the eight-century period when the books were written (Mayer, 1994). Overall, both religions prescribe positive emotions and proscribe negative emotions, and their followers are indeed more positive in their emotional expressions.

It should be noted that lexical patterns found in Tweets did not always parallel those of sacred texts. When the number of positive and negative emotion words was compared across religions rather than within each religion, participants showed a lexical pattern opposite to the sacred texts. While the Bible contained more negative and fewer positive emotion words than the Buddhist texts, Christians used more positive and less negative words than Buddhists. However, this difference was small (|*d*| < 0.2), and despite being statistically significant, it was negligible.

There could be several reasons for the discrepancy of emotional tones between the sacred texts and the receivers of the messages. Even though emotions in texts could be contagious to their readers (Kramer et al., 2014), the emotional influence of sacred texts on religious adherents might be counteracted by other behavioral or sociological factors. For example, relative to Buddhists, Christians might have more supportive social interactions, which lead to more positive emotions (Graham and Haidt, 2010). Additionally, according to the religiosity-as-social-value hypothesis (Gebauer et al., 2012), Christians in our study might be more socially valued and therefore happier than Buddhists because Christianity is the predominant religion in the United States.

Overall, our descriptive findings—linguistically, Christians appear social while Buddhists appear contemplative but comparably happy—can be interpreted from different causal directions. For example, perhaps people who are more socially active resonate more with Christianity and its activities, and people high in need for cognition are more inclined to accept and believe teachings from Buddhism. In other words, while religions as meaning systems may shape the mind and behavior of their adherents (Silberman, 2005), these adherents may also be attracted to different religions due to their predispositions (Saroglou, 2012).

Finally, our findings about religious adherents in the United States may not fully generalize to religious populations living in a society that is not Western, educated, industrialized, rich, and democratic (Henrich et al., 2010). In countries where Christianity is a minority religion, Christians may be predisposed against social conformity and not socially well-valued. As a result, Christians may not appear as social and positive compared to our study. A further cross-country comparison on these religious differences is needed in future research.

In conclusion, people with various religious affiliations can be quite different psychologically. Although Christians and Buddhists did not differ much in the number of observed positive and negative emotions, they diverged from each other in both the levels of social and cognitive tendencies. The heterogeneity between Christians and Buddhists observed in the present study suggests future studies should not treat religious effects as universal to all religious believers but instead consider religious differences.

## Author Contributions

Both authors developed the study concept, contributed to the study design, interpreted the results, and wrote the manuscript. Data collection and analysis were performed by C-YC under the supervision of T-RH.

## Conflict of Interest Statement

The authors declare that the research was conducted in the absence of any commercial or financial relationships that could be construed as a potential conflict of interest.
