# Supplementary material for: Christians and Buddhists Are Comparably Happy on Twitter: A Large-Scale Linguistic Analysis of Religious Differences in Social, Cognitive, and Emotional Tendencies
Source: Front Psychol. 2019 Feb 6;10:113. doi: 10.3389/fpsyg.2019.00113 (PMC6374623; doi:10.3389/fpsyg.2019.00113)
Supplement: Supplementary file 1 [file Table_1.DOCX]

**Type:** Supplementary Materials

**Title:** Christians and Buddhists are Comparably Happy on Twitter: A Large-Scale Linguistic Analysis of Religious Differences in Social, Cognitive, and Emotional Tendencies

**Short Title:** Christians and Buddhists are Comparably Happy

**Author Affiliation:**

Chih-Yu Chen^1^ ([chihyuchen@mil.psy.ntu.edu.tw](mailto:chihyuchen@mil.psy.ntu.edu.tw))

Tsung-Ren Huang^1,2^ ([tren@mil.psy.ntu.edu.tw](mailto:tren@mil.psy.ntu.edu.tw))

^1^ Department of Psychology, National Taiwan University

^2^ Center for Research in Econometric Theory and Applications, National Taiwan University

**Corresponding Author:**

Tsung-Ren Huang ([tren@mil.psy.ntu.edu.tw](mailto:tren@mil.psy.ntu.edu.tw))

Address: No. 1, Sec. 4, Roosevelt Rd., Taipei 10617, Taiwan

Phone: +886-2-3366-3104

**Keywords:** Religion, Christianity, Buddhism, Sociolinguistics, Social Interaction, Cognition, Emotions

**Supplementary Materials**

**Selection of Portals to Religious Users**

We selected popular religious Twitter accounts as portals to targeted religious users. This process was carried out using Followerwonk (<http://moz.com/followerwonk/bio>). First, we searched for religion-related words in the “bio” section of user accounts. We then determined if the outcome accounts were religious only and used English as the primary language of communication. For example, if an account represented both a Christian figure and an athlete, or if it had posted tweets mainly in a language other than English, it was excluded from the list of portal nominees. Among the nominees, the first two most popular accounts (i.e., accounts with the most followers) were chosen as the portals for reaching religious Twitter users.

For Christian portals, portal accounts had to contain the word “Bible” in their bio (case-insensitive). The reason for choosing “Bible” instead of “Christian” or “Jesus” was that there were many people named “Christian” or “Jésus”, especially among those with a Spanish, Portuguese, or Latin American origin, but these users were mostly not religious figures or organizations. In November 2016, the two most popular religious accounts were @Bible_Time (<http://twitter.com/Bible_Time>) and @LovLikeJesus (http://twitter.com/LovLikeJesus), with approximately 1.5 million and 1.2 million followers respectively. For Buddhist portals, accounts had to contain “Buddhist” in their bio (case-insensitive). In November 2016, the chosen portals were @thichnhathanh (<http://twitter.com/thichnhathanh>) and @Buddhism_Now (<http://twitter.com/Buddhism_Now>), with approximately 370,000 and 150,000 followers respectively.

**Sampling of Participants from Pools of Religious Users**

The four chosen portals formed one large pool of Christian users and one pool of Buddhist users. Users had to meet two criteria to be chosen as our study participants: they had to be living in the U.S. and had to have posted at least 100 English tweets by November 2016.

Because Twitter users were mostly U.S. residents and Indians, to rule out national and cultural effects, target participants included only U.S. residents. To restrict our participants to U.S. residents, we queried the ArcGIS REST API World Geocode Service (<http://developers.arcgis.com/rest/geocode/api-reference/overview-world-geocoding-service.htm>) with the “Location” information users provided on their Twitter profiles, which provided the country the user most likely lived in based on its database. Only users who listed the U.S. or any city in the U.S. as their location were eligible to participate.

Second, users had to have posted at least 100 English tweets in their last 1000 tweets in order for us to predict their age and gender. Because age and gender were highly predictive of the levels of social interaction, emotional experiences, and verbal expressions (Charles & Carstensen, 2010; Stone et al., 2010), including age and gender as control factors was crucial to our study. In a dataset collected from Facebook, Twitter, and blogs, Sap et al. (2014) could reach a predictive accuracy of .820 for age and .901 for gender using only 100 messages/tweets for each person. Thus, we sampled the first 100 English tweets posted by each user in the pools and applied the predictive lexica to infer each user’s age and gender.

To examine the effects of age and gender, we categorized age into “Under 12”, “18-24”, “25-34”, “35-44”, “45-54”, “55-64”, and “Over 65”. Limited by the penetration rate of Twitter in different age groups and in different religions (e.g., too few Buddhist users aged 12-18), only users aged 18-24, 25-34, 35-44, or 45-54 were kept as participant candidates. Finally, for each religion, we randomly sampled 1,250 people from each of the combination of age groups (18-24, 25-34, 35-44, & 45-54) and gender groups (female & male). The final sample of participants consisted of 10,000 Christian Twitter users and 10,000 Buddhist Twitter users, both evenly distributed in age and gender.

**Sampling, Preprocessing, and Processing of Participants’ Tweets**

In addition to inferring gender and age, the first 100 English tweets from each user were also used for further linguistic analyses. These tweets did not include re-tweets because re-tweets serve the function of sharing in Twitter and are not written by users who re-tweet the tweets. Before the analyses, all hyperlinks and mentions (a way to mention and notify a certain user in Twitter, in a form of @+username) were removed from each segment.

**Application of Linguistic Inquiry and Word Count (LIWC) to Tweets**

We applied LIWC (Pennebaker et al., 2015) to the tweets. LIWC categorizes words into several linguistic, psychological, and physical categories. For example, “negative emotion” contains words such as “disturbing”, “feared”, and “rigid”. In the current study, we targeted six LIWC categories: “social processes” and its closely related “personal pronouns”, “cognitive processes”, which also has the subcategory “insight”, and affective processes including “positive emotions” and “negative emotions”. LIWC computed the frequencies of the words and then the relative frequency for each category (number of target words divided by number of total words in a given text, or in our case, in 100 tweets written by each Twitter user). Each participant eventually had six scores, each representing the relative frequency of a single word category in his/her sampled tweets. These six scores were then aggregated by religion, as shown in Table S1.

| **Word Category** | **Christian** | | |  | **Buddhist** | | |
| --- | --- | --- | --- | --- | --- | --- | --- |
|  | ***N*** | **Mean** | ***SD*** |  | ***N*** | **Mean** | ***SD*** |
| **Social Interaction** | 10,000 | .13 | .04 |  | 10,000 | .12 | .03 |
| **Personal Pronouns** | 10,000 | .12 | .04 |  | 10,000 | .10 | .04 |
| **Cognitive Processes** | 10,000 | .10 | .03 |  | 10,000 | .11 | .03 |
| **Insight** | 10,000 | .02 | .01 |  | 10,000 | .03 | .01 |
| **Positive Emotions** | 10,000 | .08 | .04 |  | 10,000 | .08 | .03 |
| **Negative Emotions** | 10,000 | .02 | .01 |  | 10,000 | .02 | .02 |

**Table S1.** Descriptive statistics of relative frequencies of words in Christian and Buddhist Twitter users’ tweets.

**Selection of Religious Sacred Texts**

To investigate the possible influence of religions on their adherents, we analyzed the linguistic features of the most popular religious sacred texts. We chose Protestant Bible and Mahayana Buddhist texts for Christianity and Buddhism, respectively, because 66% of Christians in the U.S. in 2014 were Protestants (Pew Research Center, 2015) and 74% of Buddhists in the U.S. in 2010 were Mahayana Buddhists.

| **Book** | **Approximate Number of Results on Google Books** | **Book** | **Approximate Number of Results on Google Books** |
| --- | --- | --- | --- |
| Book of Genesis | 153,000 | Epistle to the Hebrews | 128,000 |
| Book of Daniel | 151,000 | Song of Solomon | 127,000 |
| Acts of the Apostles | 149,000 | Gospel of Matthew | 124,000 |
| Gospel of John | 147,000 | Gospel of Mark | 122,000 |
| Book of Job | 145,000 | Book of Isaiah | 111,000 |
| Book of Revelation | 139,000 | Gospel of Luke | 109,000 |
| Epistle to the Romans | 132,000 | Book of James | 107,000 |
| Book of Psalms | 128,000 | Book of Proverbs | 106,000 |

**Table S2.** The approximate Google Books results of the 16 chosen books in the Protestant Bible.

Because popular texts are more likely to exert a greater influence on their adherents, only the most popular sacred texts were analyzed. The index of popularity was the number of search results of each sacred text on Google Books (<http://books.google.com/>) in November 2016. For example, the “Epistle to the Romans” (132,000 results) was considered more popular than the “Gospel of Luke” (109,000 results). The inclusion criterion of popular Protestant Bible books was set as 100,000 search results. Among the 39 books in the Old Testament and 27 books in the New Testament, 16 books yielded over 100,000 results (Table S2). For Mahayana Buddhism, candidate books were those introduced by at least one dedicated Wikipedia page and yielding over 10,000 search results from Google Books (Table S3). Among the candidates, Vinaya Pitaka mainly discusses rules for bhikkhus and bhikkhunis (monks and nuns in Buddhism) and the complete collections of Pali Canonand and Sutta Pitaka were unavailable. In the end, five Buddhist texts, the Dhammapada, the Lotus Sutra, the Diamond Sutra, the Heart Sutra, and the Lankavatara Sutra, were included in our final linguistic analyses.

| **Book** | **Approximate Number of Results on Google Books** | **Book** | **Approximate Number of Results on Google Books** |
| --- | --- | --- | --- |
| Dhammapada | 176,000 | Heart Sutra | 30,400 |
| Lotus Sutra | 103,000 | Vinaya Pitaka | 29,400 |
| Pali Canon (Tipitaka) | 83,900 | Lankavatara Sutra | 18,200 |
| Diamond Sutra | 33,600 | Sutta Pitaka | 17,400 |

**Table S3.** The approximate Google Books results of the chosen Mahayana Buddhist sacred texts.

**Sources and Preprocessing of Religious Sacred Texts**

For Christian texts, we used the Modern King James Version Protestant Bible published in 1989 because 55% of survey participants who considered themselves Bible readers read the King James Version Bible. To analyze only the contents of the Bible, copyright notice, appendices, book titles (e.g., “The First Book of Moses: Called Genesis” for the first book in the Old Testament: Book of Genesis), and chapter-verse indicators (e.g., 13:1 indicating the start of chapter 13 verse 1) were removed prior to our analyses.

Similarly, we preprocessed Buddhist texts to ensure analysis of only meaningful content. For the Dhammapada translated by Acharya Buddharakkhita in 1996, its introduction, everything before chapter one, chapter titles (e.g., “Yamakavagga: Pairs”), and verse indicators (e.g., “1.” in “1. Mind precedes all mental states.”) were removed. For the Lotus Sutra translated by Kern in 1884, its Sanskrit Book title “SADDHARMA-PUNDARÎKA”, chapter indicators (e.g., “CHAPTER I” in “CHAPTER I INTRODUCTORY.”), and section indicators (e.g., “1.” in “1. Why, Mañgusrî, does this...”) were removed. For the Diamond Sutra translated by Hsuan Hua in 2002, everything before chapter one and all of the commentaries were removed. For the Heart Sutra translated by the renowned Mahayana Buddhist Thich Nhat Hanh in 2014, all text except the title “The Insight that Brings Us to the Other Shore” and the copyright notice at the end of the scripture were kept for further analyses. For the Lankavatara Sutra translated by Daisetsu Teitaro Suzuki in 1932, all chapter indices, titles (e.g., “Chapter I Discrimination”), and page numbers were removed.

**Unit of Comparison Between Religions for Twitter and Religious Texts**

For a parallel presentation of LIWC results for Twitter data and religious sacred texts, the unit of comparison should be relatively equal. For Twitter data, the unit of linguistic analysis was a user who had exactly 100 tweets (~1,140 words). Thus, we divided each book in the Bible and each Buddhist text into excerpts of 1,000 words. Excerpts with fewer than 348 words, namely, the total number of words in the Heart Sutra, were excluded from further analyses because smaller excerpts could greatly distort the mean of relative frequencies for all texts. For example, if an excerpt contained only 3 words “I am sad”, the relative frequency of negative emotion words would be .33, which was significantly higher than what we observed in tweets and other text excerpts with longer content. After application of LIWC, each excerpt would lead to six scores representing the relative frequencies of words in the six categories of interest: social processes, personal pronouns, cognitive processes, insight, positive emotions, and negative emotions. These six scores were then aggregated by religion, as shown in Table S4.

| **Word Category** | **Popular Bible Books** | | |  | **Buddhist Sacred Texts** | | |  |
| --- | --- | --- | --- | --- | --- | --- | --- | --- |
|  | ***N*** | **Mean** | ***SD*** |  | ***N*** | **Mean** | ***SD*** | |
| **Social Interaction** | 305 | .18 | .04 |  | 144 | .10 | .03 | |
| **Personal Pronouns** | 305 | .16 | .04 |  | 144 | .06 | .02 | |
| **Cognitive Processes** | 305 | .08 | .03 |  | 144 | .12 | .04 | |
| **Insight** | 305 | .01 | .01 |  | 144 | .03 | .02 | |
| **Positive Emotions** | 305 | .03 | .02 |  | 144 | .05 | .02 | |
| **Negative Emotions** | 305 | .02 | .01 |  | 144 | .02 | .02 | |

**Table S4.** Descriptive statistics of relative frequencies of words in the Protestant Bible and Mahayana Buddhist sacred text excerpts.

**Distributions of the Six Categories by Age and Gender**

Distributions of the six categories of interest are modulated by age and gender, as shown in Figs. S1-S6.

**Fig. S1.** Distributions of the relative frequencies of (**A**) social words and (**B**) personal pronouns in tweets from Twitter users of four age groups: 18-24, 25-34, 35-44, and 45-54 years of age. The younger religious Twitter users were, the more personal pronouns they used in tweets (*Mean Square* = 2.15, *F*(3,19996) = 1801.34, *p* < .001, *ω^2^* = .21, *r* = -.47). Those aged 45-54 also used social-interaction words the least compared with their counterparts in other age groups (*Mean Square* = .02, *F*(3,19996) = 13.95, *p* < .001, *ω^2^* = .00, *r* = -.02).

**Fig. S2.** Distributions of the relative frequencies of (**A**) social words and (**B**) personal pronouns in tweets of female and male Twitter users. Female Twitter users used more social words and personal pronouns than male Twitter users (social words: Kolmogorov-Smirnov test’s D statistic [*KS D*] = .12, *p* < .001, *Cohen’s d* 95% *CI*: [.25, .31]; personal pronouns: *KS D* = .17, *p* < .001, *Cohen’s d* 95% *CI*: [.38, .44]).

**Fig. S3.** Distributions of the relative frequencies of (**A**) cognitive words and (**B**) insight words in tweets of Twitter users in four age groups: 18-24; 25-34; 35-44; and 45-54 years of age. The younger religious Twitter users were, the more cognitive words they used in tweets (*Mean Square* = 1.28, *F*(3,19996) = 1434.50, *p* < .001, *ω^2^* = .18, *r* = -.42). Those aged 45-54 also used insight-related words the least compared with counterparts in other age groups (*Mean Square* = .01, *F*(3,19996) = 56.32, *p* < .001, *ω^2^* = .01, *r* = -.05).

**Fig. S4.** Distributions of the relative frequencies of (**A**) cognitive words and (**B**) insight words in tweets of female and male Twitter users. Female Twitter users did not differ greatly in the amount of cognitive words and insight words used compared to male Twitter users (cognitive words: *KS D* = .05, *p* < .001, *Cohen’s d* 95% *CI*: [.04, .10]; insight: *KS D* = .04, *p* < .001, *Cohen’s d* 95% *CI*: [.04, .10]).

**Fig. S5.** Distributions of the relative frequencies of (**A**) positive emotion words and (**B**) negative emotion words in tweets of Twitter users in four age groups: 18-24, 25-34, 35-44, and 45-54 years of age. The younger religious Twitter users were, the more negative emotion words they used in tweets (*Mean Square* = .22, *F*(3,19996) = 1221.50, *p* < .001, *ω^2^* = .16, *r* = -.39). Religious Twitter users 18-24 years of age were also the least positive in verbal emotional expressions (*Mean Square* = .37, *F*(3,19996) = 292.61, *p* < .001, *ω^2^* = .04, *r* = .20).

**Fig. S6.** Distributions of the relative frequencies of (**A**) positive emotion words and (**B**) negative emotion words in tweets of female and male Twitter users. Female Twitter users used more positive emotion words than male Twitter users (*KS D* = .15, *p* < .001, *Cohen’s d* 95% *CI*: [.24, .30]) The two genders did not differ much in the amount of verbal expression of negative emotions (*KS D* = .03, *p* = .0009, *Cohen’s d* 95% *CI*: [-.06,
.00]).

**References**

Charles, S. T., & Carstensen, L. L. (2010). Social and emotional aging. *Annual Review of Psychology*, *61*, 383–409. https://doi.org/10.1146/annurev.psych.093008.100448

Goff, P., Farnsley, A. E., & Thuesen, P. J. (2014). *The Bible in American life*. Retrieved from <http://www.raac.iupui.edu/files/2713/9413/8354/Bible_in_American_Life_Report_March_6_2014.pdf>

Grammich, C., Hadaway, K., Houseal, R., Jones, D. E., Krindatch, A., Stanley, R., & Taylor, R. H. (2012). *2010 U.S. religion census: Religious congregations & membership study*. Retrieved from <http://www.rcms2010.org/index.php>.

Pennebaker, J. W., Booth, R. J., Boyd, R. L., & Francis, M. E. (2015). Linguistic Inquiry and Word Count: LIWC2015 [Computer Program]. Austin, TX: Pennebaker Conglomerates. Retrieved from <http://liwc.wpengine.com/>.

Pew Research Center (2015). *America’s changing religious landscape: Christians decline sharply as share of population; unaffiliated and other faiths continue to grow*. Washington, D.C., U.S. Retrieved from http://www.pewforum.org/2015/05/12/americas-changing-religious-landscape/.

Sap, M., Park, G., Eichstaedt, J. C., Kern, M. L., Stillwell, D., Kosinski, M., … Schwartz, H. A. (2014). Developing age and gender predictive lexica over social media. In *The 2014 Conference on Empirical Methods In Natural Language Processing* (pp. 1146–1151).

Statista. (2016). Number of active Twitter users in leading markets as of May 2016 (in millions) [Web Page]. Retrieved November 10, 2016, from <https://www.statista.com/statistics/242606/number-of-active-twitter-users-in-selected-countries/>

Stone, A. A., Schwartz, J. E., Broderick, J. E., & Deaton, A. (2010). A snapshot of the age distribution of psychological well-being in the United States. *Proceedings of the National Academy of Sciences of the United States of America*, *107*(22), 9985–9990. https://doi.org/10.1073/pnas.1003744107
